# Supplementary material for: Exploring the link between coffee matrix microstructure and flow properties using combined X-ray microtomography and smoothed particle hydrodynamics simulations
Source: Sci Rep. 2023 Sep 29;13:16374. doi: 10.1038/s41598-023-42380-y (PMC10541431; doi:10.1038/s41598-023-42380-y)
Supplement: Supplementary file 1 — Supplementary Information. [file 41598_2023_42380_MOESM1_ESM.pdf]

Supplementary Material: Exploring the link between coffee matrix microstructure and flow properties using combined X-ray microtomography and smoothed particle hydrodynamics simulations

Chaojie Mo*[1,2], Richard Johnston[3], Luciano Navarini[4], Furio Suggi Liverani[4], and Marco Ellero[1,5,6]

[1]Basque Center for Applied Mathematics (BCAM), Alameda de Mazarredo 14, 48009 Bilbao, Spain
[2]Aircraft and Propulsion Laboratory, Ningbo Institute of Technology, Beihang University, Ningbo 315100, P. R. China
[3]Faculty of Science and Engineering, Swansea University, Swansea, SA1 8EN, UK
[4]Illycaffè S.p.A, Via Flavia 110, Trieste 34147, Italy
[5]Zienkiewicz Centre for Computational Engineering (ZCCE), Swansea University, Bay Campus, Swansea SA1 8EN, UK
[6]IKERBASQUE, Basque Foundation for Science, Calle de María Díaz de Haro 3, 48013 Bilbao, Spain

1 Smoothed Particle Hydrodynamics formalism

In our model the flow is governed by the Navier-Stokes equations. To solve them, we employ the smoothed particle hydrodynamics (SPH) method [1, 2], which is a particle-based hydrodynamics approach. SPH is derived through a Lagrangian discretisation of the Navier-Stokes equations. We employ an SPH version which conserves angular momentum [3], as it can be crucial for some problems [4–6]. In SPH, each particle can be considered as a small fluid volume (or Lagrangian discretisation point) characterised by a position $\boldsymbol{r}_i$, velocity $\boldsymbol{v}_i$, and mass m_i. In addition, each SPH particle possesses a spin angular velocity $\boldsymbol{\psi}_i$ and moment of inertia I_i introduced for the enforcement of angular momentum conservation [3].

SPH particles i and j interact through three pairwise forces, including conservative $\boldsymbol{F}_{ij}^C$, dissipative $\boldsymbol{F}_{ij}^D$, and rotational $\boldsymbol{F}_{ij}^R$ forces given by

$$
\begin{aligned}
\boldsymbol{F}_{ij}^C &= \left(\frac{P_i}{d_i^2} + \frac{P_j}{d_j^2}\right) F_{ij}\boldsymbol{r}_{ij}, \\
\boldsymbol{F}_{ij}^D &= -\gamma_{ij}\left[\boldsymbol{v}_{ij} + (\boldsymbol{e}_{ij}\cdot\boldsymbol{v}_{ij})\boldsymbol{e}_{ij}\right], \\
\boldsymbol{F}_{ij}^R &= -\gamma_{ij}\frac{\boldsymbol{r}_{ij}}{2}\times(\boldsymbol{\psi}_i + \boldsymbol{\psi}_j),
\end{aligned}
\tag{1}
$$

where $\boldsymbol{r}_{ij} = \boldsymbol{r}_i - \boldsymbol{r}_j$, $\boldsymbol{v}_{ij} = \boldsymbol{v}_i - \boldsymbol{v}_j$, and $\boldsymbol{e}_{ij} = \boldsymbol{r}_{ij}/r_{ij}$. Particle number density d_i is computed as $d_i = \sum_j W_{ij}$ using a smoothing kernel function $W_{ij} = W(r_{ij})$ that vanishes beyond a cutoff radius r_c and defines a non-negative function F_{ij} through the equation $\nabla_i W_{ij} = -\boldsymbol{r}_{ij}F_{ij}$. Then, particle mass density is given by $\rho_i = m_i d_i$. The pressure P_i is determined by the equation of state (EoS) $P_i = P_0(d_i/d_0)^\nu - P_\mathrm{b}$, where d_0 is the average number density, P_0 and ν are parameters controlling the sound speed $c = \sqrt{P_0\nu/d_0}$, and P_b relates to the background pressure. Furthermore, γ_{ij} is a force amplitudes defined as

$$
\gamma_{ij} = \frac{20\eta}{7}\frac{F_{ij}}{d_i d_j}, \tag{2}
$$

where η is the fluid dynamic viscosity.

*cmo@bcamath.org

Table 1: Basic parameters for the SPH simulations.

**Parameters**	**Values**	**Dimensions**	**Values in SI units**
Cutoff radius r_c	1.0	L	51.7μm
Mass density ρ	30.0	ML^{-3}	1000kg/m
Dynamic viscosity η	2	$ML^{-1}T^{-1}$	$1\text{mPa}\cdot\text{s}$
Average number density d_0	30	L^{-3}	217490mm^{-3}
SPH particle mass m	1	M	4.60×10^{-12}kg
Moment of inertial of SPH particles I	1	ML^2	$1.23\times10^{-20}\text{kg}\cdot\text{m}^2$
P_0 in the EoS	6400	$ML^{-1}T^{-2}$	18.0kPa
Hydrostatic pressure $P_0 - P_\text{b}$	80	$ML^{-1}T^{-2}$	225Pa
Exponent in the EoS ν	7	1	7
Time step Δt	0.001	T	0.18ms

The evolution of particle positions, translational and angular velocities is obtained by integration of the following equations of motion

$$\begin{gathered}\dot{\boldsymbol{r}}_i = \boldsymbol{v}_i,\\ m_i\dot{\boldsymbol{v}}_i = \sum_j \boldsymbol{F}_{ij} = \sum_j(\boldsymbol{F}^C_{ij} + \boldsymbol{F}^D_{ij} + \boldsymbol{F}^R_{ij}),\\ \dot{\boldsymbol{\psi}}_i = \frac{1}{2I_i}\sum_j \boldsymbol{r}_{ij}\times\boldsymbol{F}_{ij},\end{gathered} \tag{3}$$

using the velocity-Verlet algorithm [7].

In this work, the smoothing kernel is represented by the quintic spline kernel function [8]

$$W(q) = w_0\begin{cases}(3-q)^5 - 6(2-q)^5 + 15(1-q)^5, & 0\le q<1\\ (3-q)^5 - 6(2-q)^5, & 1\le q<2\\ (3-q)^5, & 2\le q<3\\ 0, & q\ge 3\end{cases} \tag{4}$$

where $q = r/h$ and $w_0 = 1/(120\pi h^3)$ in three dimensions (3-D), $w_0 = 7/(478\pi h^2)$ in two dimensions (2-D), and h is the smoothing length $h = r_c/3$.

The basic parameters used in our simulations are summarized in table 1.

2 Enlarged vertical sections of the microCT imaging for different powders

We show enlarged vertical digital sections of the microCT/XRM imaging data for different powders in Fig. 1 (type E), Fig. 2 (type H), Fig. 3 (type M), and Fig. 4 (type F). Both the coffee grains and the plastic/metal structure skeletons of the capsules are clearly visible in these images. It is also discernible that the coffee particles get coarser from type E to type F.

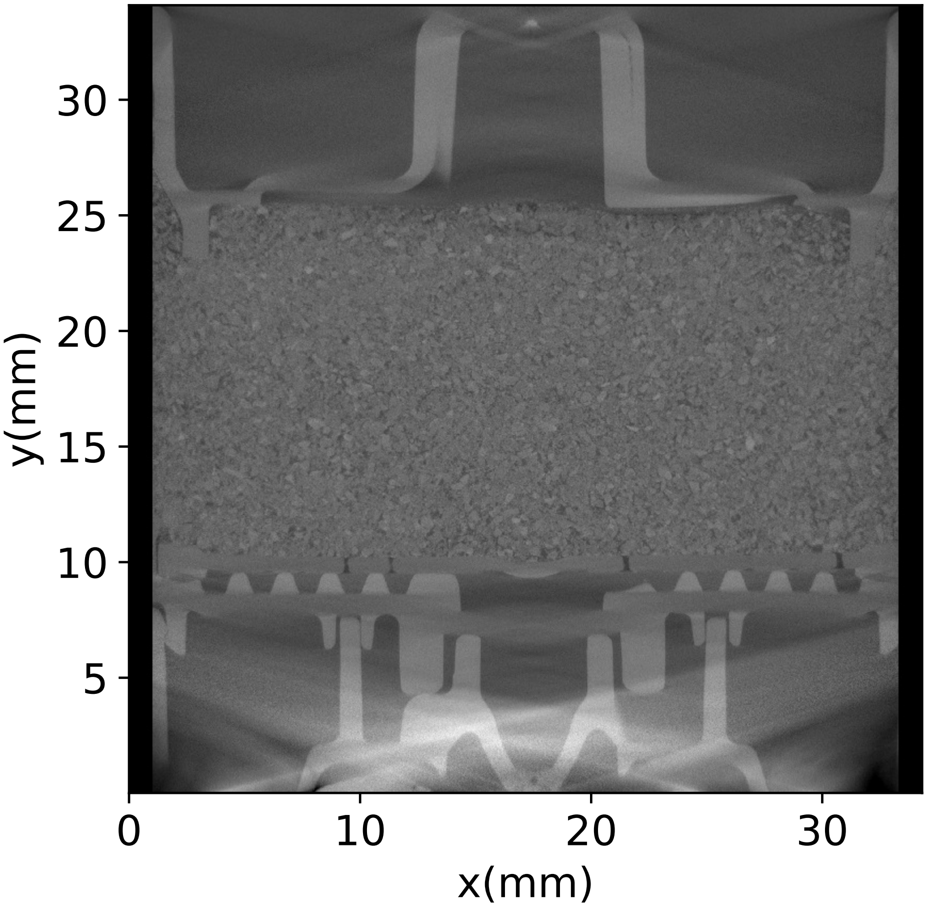

Figure 1: An enlarged vertically-cut section of the microCT imaging for the type E coffee powder.

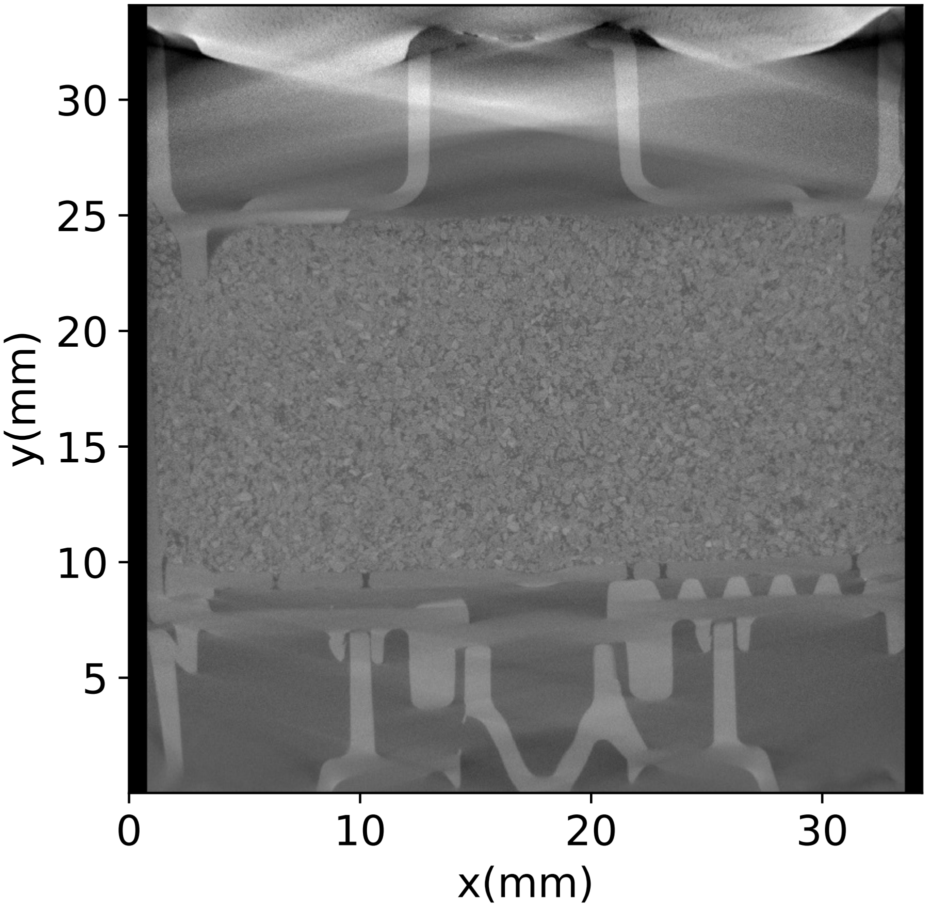

Figure 2: An enlarged vertically-cut section of the microCT imaging for the type H coffee powder.

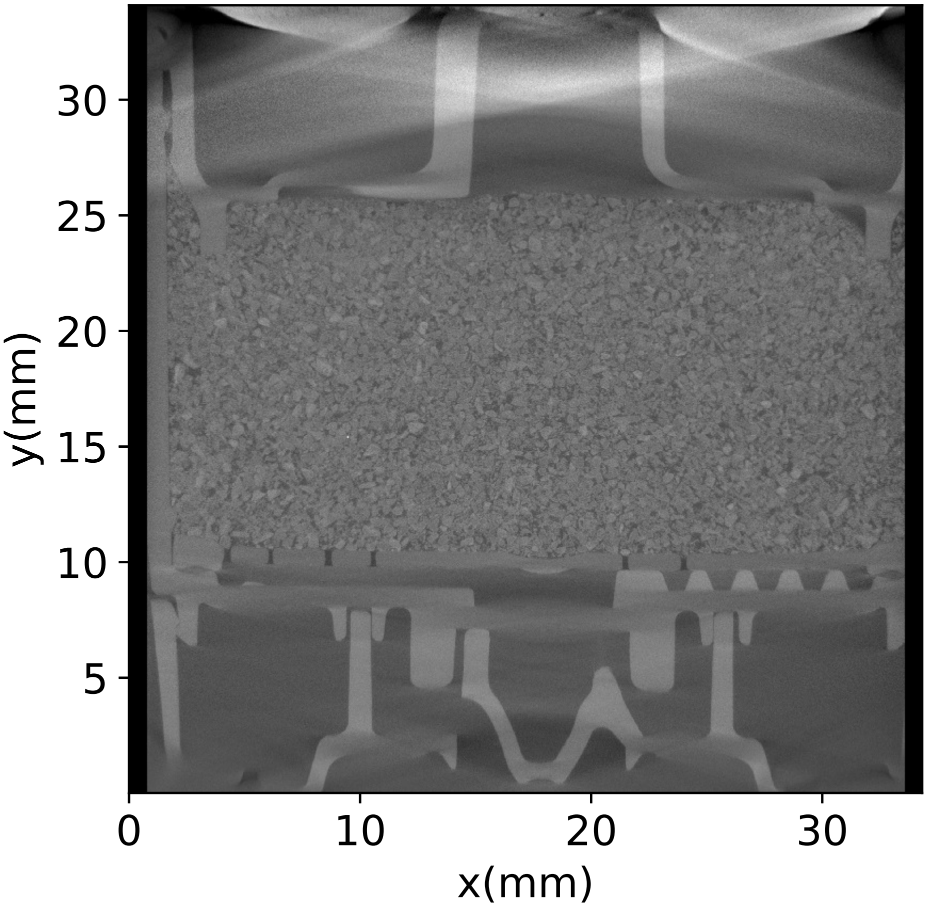

Figure 3: An enlarged vertically-cut section of the microCT imaging for the type M coffee powder.

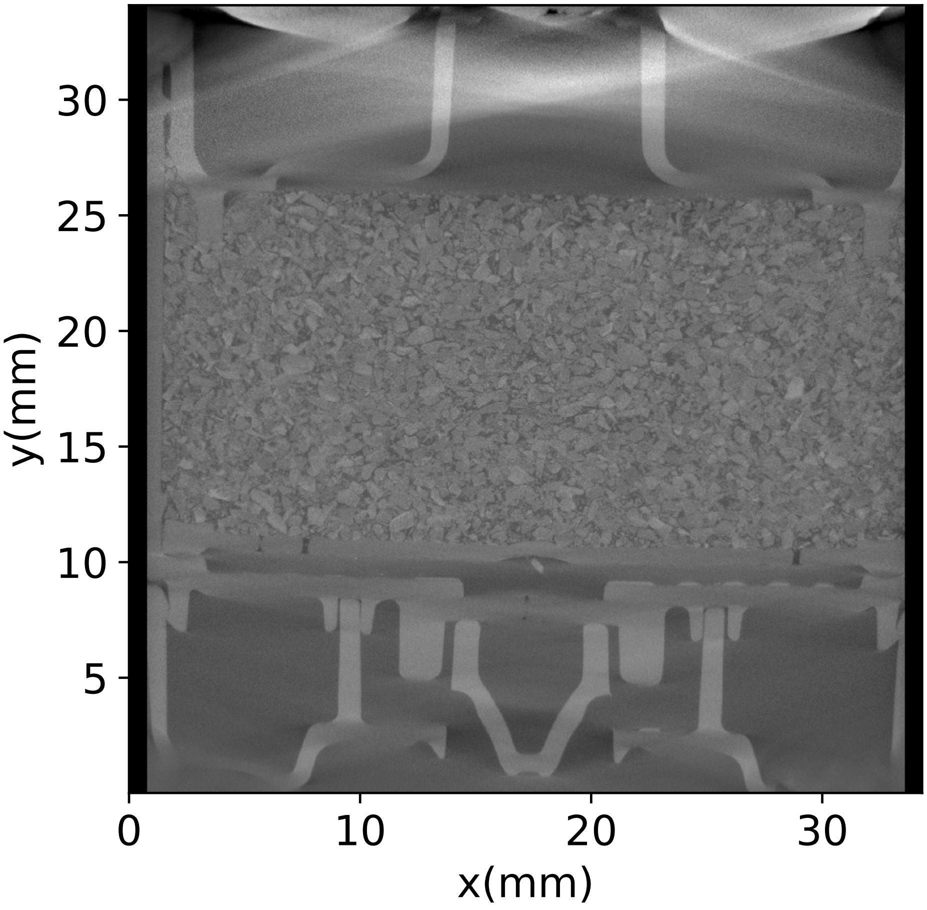

Figure 4: An enlarged vertically-cut section of the microCT imaging for the type F coffee powder.

Bibliography

[1] J. J. Monaghan. Smoothed particle hydrodynamics. *Annu. Rev. Astron. Astrophys.*, 30:543–574, 1992.

[2] Marco Ellero, Mar Serrano, and Pep Español. Incompressible smoothed particle hydrodynamics. *Journal of Computational Physics*, 226(2):1731–1752, 2007.

[3] K. Müller, D. A. Fedosov, and G. Gompper. Smoothed dissipative particle dynamics with angular momentum conservation. *J. Comp. Phys.*, 281:301–315, 2015.

[4] X. Y. Hu and N. A. Adams. Angular-momentum conservative smoothed particle dynamics for incompressible viscous flows. *Phys. Fluids*, 18:101702, 2006.

[5] I. O. Götze, H. Noguchi, and G. Gompper. Relevance of angular momentum conservation in mesoscale hydrodynamics simulations. *Phys. Rev. E*, 76:046705, 2007.

[6] Adolfo Vázquez-Quesada and Marco Ellero. Rheology and microstructure of non-colloidal suspensions under shear studied with smoothed particle hydrodynamics. *Journal of Non-Newtonian Fluid Mechanics*, 233:37–47, 2016.

[7] M. P. Allen and D. J. Tildesley. *Computer simulation of liquids*. Clarendon Press, New York, 1991.

[8] Marco Ellero and R. I. Tanner. SPH simulations of transient viscoelastic flows at low Reynolds number. *Journal of Non-Newtonian Fluid Mechanics*, 132(1-3):61–72, 2005.
